# Eicosapentaenoic acid but not docosahexaenoic acid restores skeletal muscle mitochondrial oxidative capacity in old mice

**DOI:** 10.1111/acel.12352

**Published:** 2015-05-25

**Authors:** Matthew L Johnson, Antigoni Z Lalia, Surendra Dasari, Maximilian Pallauf, Mark Fitch, Marc K Hellerstein, Ian R Lanza

**Affiliations:** 1 Division of Endocrinology and Metabolism, Mayo Clinic College of MedicineRochester, MN, USA; 2 Division of Biomedical Statistics and Informatics, Mayo Clinic College of MedicineRochester, MN, USA; 3 Department of Nutritional Sciences and Toxicology, University of California BerkeleyBerkeley, CA, USA

**Keywords:** aging, docosahexaenoic acid, eicosapentaenoic acid, mitochondria, omega 3, proteomics, sarcopenia

## Abstract

Mitochondrial dysfunction is often observed in aging skeletal muscle and is implicated in age-related declines in physical function. Early evidence suggests that dietary omega-3 polyunsaturated fatty acids (n-3 PUFAs) improve mitochondrial function. Here, we show that 10 weeks of dietary eicosapentaenoic acid (EPA) supplementation partially attenuated the age-related decline in mitochondrial function in mice, but this effect was not observed with docosahexaenoic acid (DHA). The improvement in mitochondrial function with EPA occurred in the absence of any changes in mitochondrial abundance or biogenesis, which was evaluated from RNA sequencing, large-scale proteomics, and direct measurements of muscle mitochondrial protein synthesis rates. We find that EPA improves muscle protein quality, specifically by decreasing mitochondrial protein carbamylation, a post-translational modification that is driven by inflammation. These results demonstrate that EPA attenuated the age-related loss of mitochondrial function and improved mitochondrial protein quality through a mechanism that is likely linked with anti-inflammatory properties of n-3 PUFAs. Furthermore, we demonstrate that EPA and DHA exert some common biological effects (anticoagulation, anti-inflammatory, reduced FXR/RXR activation), but also exhibit many distinct biological effects, a finding that underscores the importance of evaluating the therapeutic potential of individual n-3 PUFAs.

## Introduction

Maintenance of skeletal muscle health across the lifespan is critical for physical function and well-being. Sarcopenia, the loss of muscle mass and function with age, is compounded by some reports of age-related derangements in mitochondrial physiology (Lanza *et al*., 2012), which includes decreased mitochondrial capacity (Short *et al*., 2005), decreased mitochondrial abundance (Conley *et al*., 2000), decreased mitochondrial protein expression (Lanza *et al*., 2008), and mRNA transcripts of nuclear and mitochondrial encoded genes (Short *et al*., 2005). Furthermore, mitochondrial aging is associated with increased oxidative stress (Hepple *et al*., 2008; Lanza *et al*., 2012) and decreased protein quality (Feng *et al*., 2008; Lanza *et al*., 2012). It is important to highlight that aforementioned effects of age on mitochondrial physiology are not universally accepted as many carefully conducted studies do not find mitochondrial abnormalities in aged humans (Hütter *et al*., 2007; Gouspillou *et al*., 2014) or rodents (Picard *et al*., 2011; Siegel *et al*., 2013) Nevertheless, the age-related changes in mitochondrial function evident under some conditions, together with changes in central hemodynamics (Proctor & Joyner, 1997), are believed to contribute to the steady decline in maximal oxygen uptake (VO_2_ peak) with age.

Potential countermeasures to prevent or delay sarcopenia and age-related metabolic derangements are of great interest. We previously demonstrated that exercise (Lanza *et al*., 2008) and life-long caloric restriction (Lanza *et al*., 2012) delay mitochondrial aging in skeletal muscle. Exercise in particular has been shown to be a highly effective strategy for increasing muscle mass (Leenders *et al*., 2013), muscle protein synthesis (Balagopal *et al*., 1997; Hasten *et al*., 2000), mitochondrial content (Jubrias *et al*., 2001; Menshikova *et al*., 2006), and whole-body VO_2_ peak (Coggan *et al*., 1992) in humans. However, in many cases, these adaptations are blunted, slower to occur, and more rapidly lost after exercise in older adults (Coggan *et al*., 1992; Balagopal *et al*., 2001; Short *et al*., 2003; Menshikova *et al*., 2006). Furthermore, for many individuals, exercise is impossible due to physical limitations or other health-related contraindications. These individuals require alternative therapeutic approaches to maintain healthy, functional muscle.

There is accumulating evidence that the omega-3 polyunsaturated fatty acids (n-3 PUFAs) eicosapentaenoic acid (EPA) and docosahexaenoic acid (DHA) may be effective nonpharmacological therapeutics to improve mitochondrial physiology in aging skeletal muscle. A recent study in humans showed that both EPA and DHA are incorporated into mitochondrial membranes and increase the sensitivity of mitochondria for ADP (Herbst *et al*., 2014). Furthermore, we previously reported that fish oil supplementation increased mRNA expression of *ppargc1a* and *nrf1*, two important transcriptional regulators of mitochondrial biogenesis (Lanza *et al*., 2013). Others also demonstrated in C6 glioma cells that n-3 PUFAs are ligands for receptors that activate PGC-1α (Desvergne & Wahli, 1999), leading to increased expression of PGC-1α, transcription factor-A mitochondrial (TFAM), cytochrome c oxidase, and increased the mitochondrial membrane potential (Jeng *et al*., 2009). Taken together, these studies provide promising early evidence that n-3 PUFAs may stimulate mitochondrial biogenesis, but no studies have been conducted to determine whether dietary n-3 PUFAs can prevent or reverse impairments in muscle mitochondrial content or function in the context of aging.

The primary purpose of the current study was to determine the influence of dietary n-3 PUFAs on mitochondrial content and function in skeletal muscle of young and older mice. Here, we studied the independent effects of EPA and DHA on skeletal muscle mitochondrial physiology because although both are found in fish oil, they are known to have distinct biological effects (Jeng *et al*., 2009) yet are rarely studied independently. Here, we show that 10 weeks of EPA supplementation partially restores mitochondrial oxidative capacity and bioenergetic efficiency in aging skeletal muscle, but DHA does not. Mechanistic insight into this finding is provided from large-scale quantitative proteomics, RNA sequencing, specific measurements of skeletal muscle protein synthesis, and protein post-translational modifications. Using these complementary methods, we find that EPA partially restores oxidative capacity in older mice without increasing mitochondrial abundance or biogenesis. Rather, our data indicate that EPA improves mitochondrial function through its influence on muscle protein quality, specifically by decreasing protein carbamylation, a post-translational modification that is driven by inflammation and interferes with protein function (Wang *et al*., 2007).

## Results

### Eicosapentaenoic acid improves mitochondrial function in old mice

**Fig 1:**
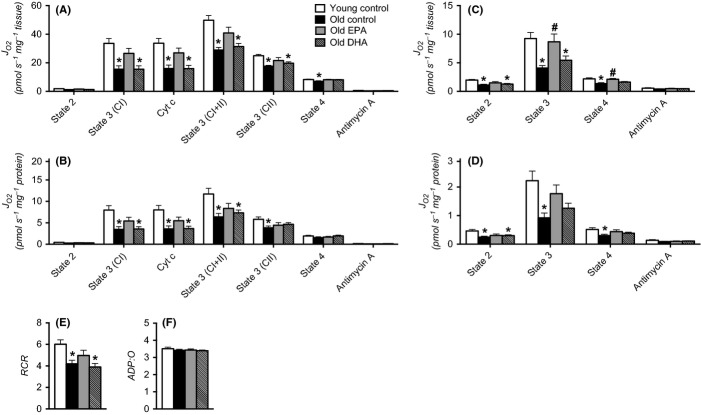
Eicosapentaenoic acid restores muscle mitochondrial function in aged mice (A,B) Isolated mitochondria were respired with substrates targeting complex I (CI), complex I+II (CI+II), and complex II (CII). Mitochondrial membrane integrity was assessed by the addition of cytochrome c (Cyt c). Nonmitochondrial oxygen consumption was measured in the presence of antimycin A (AA). (C,D) Respiration rates were also measured using palmitoyl-carnitine substrates. Respiration rates were expressed per tissue wet (A,C) and mitochondrial protein content (B,D). Respiratory control ratio (RCR, state 3/state 4) and phosphorylation efficiency (ADP:O) were measured in isolated mitochondria (E,F). Bars represent means ± SEM. *, significant statistical differences from young control (*P* < 0.05, Tukey’s HSD). ^#^, significant statistical differences from old control (*P* < 0.05, Tukey’s HSD). *n* = 8 per group.

Skeletal muscle mitochondrial oxidative capacity, evaluated from state 3 respiration in mitochondria isolated from quadriceps muscle, was lower in old mice compared with young mice in the control fed conditions (Fig.1). This age-related decline in mitochondrial capacity was observed when respiration was expressed per wet weight of muscle tissue (Fig.1A), indicating a reduction in whole-muscle oxidative capacity, and when normalized to mitochondrial protein (Fig.1B), indicating an age-related reduction in the intrinsic function of mitochondria. The addition of exogenous cytochrome c did not potentiate respiration, indicating that the integrity of the outer mitochondrial membrane was intact following isolation of the organelles. The reduction in mitochondrial capacity of old mice was evident under experimental conditions where substrates were provided through respiratory chain complex I, complex I and II together, and complex II (Fig.1A,B). Furthermore, state 3 respiration using palmitoyl carnitine and malate as substrates to evaluate lipid oxidative capacity was also significantly reduced in old mice under control conditions (Fig.1C,D). Eicosapentaenoic acid partially reversed the effect of aging on state 3 respiration rates following 10 weeks of supplementation for all substrate combinations (Fig.1A–D). In contrast, DHA supplementation in old mice did not attenuate the age-related decline in state 3 respiration (Fig.1A–D). Young mice that received EPA or DHA showed no change in mitochondrial capacity compared with young control mice (Fig. S1).

In addition to the age-related decrease in mitochondrial capacity, there was a significant decrease in mitochondrial efficiency (i.e., increased proton leak), evident from an age-related decrease in the respiratory control ratio (RCR, Fig.1E). Similar to state 3 respiration, RCR was partially restored by EPA in old mice, but not in DHA-treated mice (Fig.1E). The phosphorylation efficiency (ADP:O) was unchanged by age or supplementation with EPA or DHA (Fig.1F). Young mice that received EPA and DHA showed similar coupling efficiency as young control mice (Fig. S1). Altogether, these results show that the age-related decline in muscle mitochondrial capacity and efficiency is attenuated by dietary supplementation with EPA, but not DHA.

### Eicosapentaenoic acid does not restore age-related reductions in mitochondrial protein abundance

**Fig 2:**
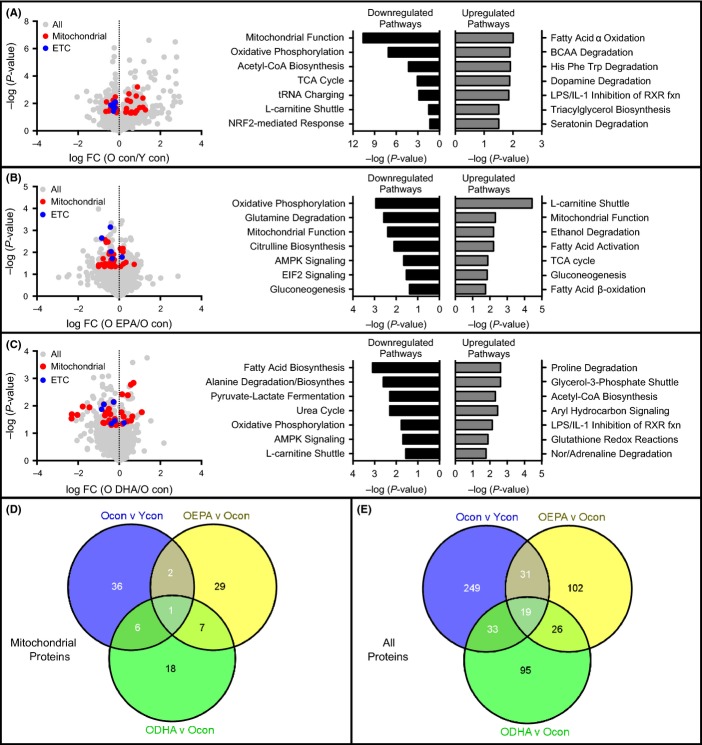
Eicosapentaenoic acid (EPA) and docosahexaenoic acid (DHA) do not restore age-related reductions in mitochondrial content of skeletal muscle volcano plots of differentially expressed muscle proteins in old vs. young control mice (A), EPA-treated old versus old control (B), and (C) DHA-treated old versus old control measured by mass spectrometry. The *x*-axis represents the log fold change (FC), while *y*-axis represents the –log *P*-value for each protein. Mitochondrial proteins are colored in red. Electron transport chain (ETC.) proteins are colored blue. Ingenuity pathway analysis was performed using mitochondrial proteins, and the top canonical pathways are given. Histogram bars represent *P* values for each pathway using either exclusively upregulated proteins (gray bars) or exclusively downregulated proteins (black bars). (D,E) Venn diagrams showing differentially expressed mitochondrial proteins (D) or all proteins (E).

We next determined whether the attenuation of mitochondrial dysfunction in EPA-treated old mice could be explained by increased abundance of mitochondrial proteins in skeletal muscle. We performed a large-scale, untargeted proteomics survey by mass spectrometry (Lanza *et al*., 2012). Of the 2901 proteins identified, 450 were mitochondrial proteins, and 45 of these mitochondrial proteins were differentially expressed in young and old control mice (Fig.2A,D). There were 27 mitochondrial proteins that were downregulated in old mice, and ingenuity pathway analysis revealed that these proteins were largely involved in mitochondrial energetics (Fig.2A). There were 18 mitochondrial proteins that were upregulated with age, which were primarily involved in amino acid degradation (Fig.2A). To evaluate the effects of age on mitochondrial content, we compared the abundance of inner membrane-bound electron transport chain (ETC) proteins in old and young mice. There were 7 ETC proteins that were significantly downregulated in old mice, including subunits of complex I, complex III, and ATP synthase (Fig.2A). We then examined which mitochondrial proteins were differentially expressed between old mice that were fed control diets or diets enriched with EPA or DHA. There were 39 mitochondrial proteins that were differentially expressed between old control and old EPA-treated (Fig.2B,D) and 32 mitochondrial proteins that were differentially expressed between old control and old DHA-treated mice (Fig.2C,D). Of these proteins, only three for EPA and seven for DHA were common to those that were differentially expressed between young and old control mice (Fig.2D). For both EPA and DHA, the majority of the mitochondrial proteins, particularly those involved in energy provision, were downregulated compared with control fed old mice. A complete list of proteins included in each canonical pathway is provided in Table S1 (Supporting information). Taken together, the mitochondrial proteomic data indicate that neither EPA nor DHA attenuated the age-related decline in mitochondrial protein content; a finding that is counterintuitive given the improvement in mitochondrial function after EPA supplementation.

### Eicosapentaenoic acid does not stimulate mitochondrial biogenesis in aged skeletal muscle

**Fig 3:**
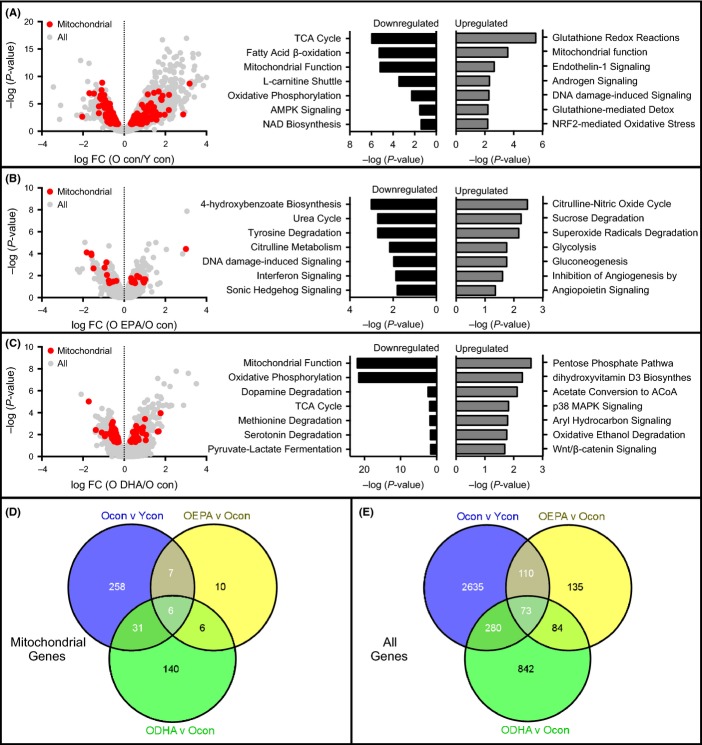
Eicosapentaenoic acid (EPA) and docosahexaenoic acid (DHA) do not stimulate mitochondrial biogenesis in aged skeletal muscle volcano plots of differentially expressed genes in old vs. young control mice (A), EPA-treated old versus old control (B), and (C) DHA-treated old versus old control measured by RNA sequencing. The *x*-axis represents the log fold change, while *y*-axis represents the –log *P*-value for each gene. Mitochondrial genes are colored in red. Ingenuity pathway analysis was performed using mitochondrial-related genes, and the top canonical pathways are given. Histogram bars represent p values for each pathway using either exclusively upregulated proteins (gray bars) or exclusively downregulated proteins (black bars). (D,E) Venn diagrams showing differentially expressed mitochondrial genes (D) or all genes (E).

Although we find no evidence after 10 weeks of supplementation that EPA or DHA increases mitochondrial abundance in muscle, there is precedent literature to suggest that n-3 PUFAs activate PGC-1α and downstream transcription factors involved in mitochondrial biogenesis (Desvergne & Wahli, 1999; Lanza *et al*., 2013). It is therefore difficult to ignore the possibility that n-3 PUFAs may stimulate mitochondrial biogenesis without changing mitochondrial abundance (i.e., increased turnover of the organelle). To evaluate mitochondrial biogenesis, we first used RNA sequencing for whole-genome transcript profiling to determine whether EPA or DHA produced a gene expression profile consistent with mitochondrial biogenesis. There were 302 mitochondrial-related genes that were differentially expressed in old control and young control mice (Fig.3A,D). Of these mitochondrial genes, 165 were downregulated and 137 were upregulated in old compared with young mice (Fig.3A). Similar to the proteomics data, the majority of the downregulated genes were involved in energy metabolism, whereas upregulated genes were related to oxidative stress (Fig.3A). EPA upregulated 17 mitochondrial genes and downregulated 12 mitochondrial genes compared with old controls (Fig.3B), none of which were linked with canonical pathways related to oxidative phosphorylation. DHA upregulated 35 mitochondrial genes and downregulated 148 mitochondrial genes compared with old controls (Fig.3C), with the majority of downregulated genes being linked with mitochondrial energetics. In sum, there are clear transcriptional patterns consistent with impaired mitochondrial function in old mice that is not reversed by EPA treatment. Furthermore, DHA supplementation features transcriptional signals consistent with further downregulation of mitochondrial oxidative capacity. Table S2 gives the genes involved in each pathway given in Fig.3.

**Fig 4:**
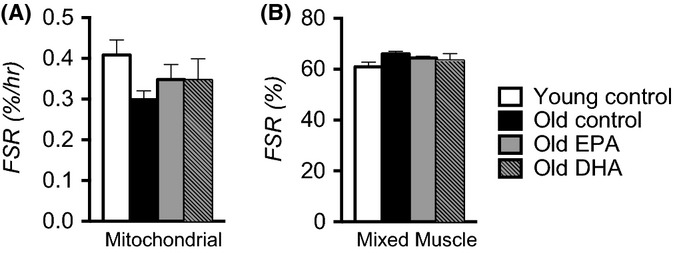
Eicosapentaenoic acid (EPA) and docosahexaenoic acid (DHA) do not stimulate protein synthesis in aged skeletal muscle fractional synthesis rates of mitochondrial proteins were measured *in vivo* from the rate of incorporation of ^13^C_6_ phenylalanine and mass spectrometry (A). The overall mixed muscle protein synthesis rate was measured by long-term labeling with deuterium oxide (B). Bars represent means ± SEM. *n* = 8 per group.

Although we found no transcriptional evidence of mitochondrial biogenesis with EPA or DHA supplementation, we further confirmed this with direct measurements of *in vivo* mitochondrial protein synthesis rates in these animals. Old mice exhibited approximately 25% lower fractional synthesis rates (FSR) of mitochondrial proteins compared with young control mice (Fig.4A), although this did not reach statistical significance. Importantly, neither EPA nor DHA increased mitochondrial protein synthesis in old mice (Fig.4A) or in young mice (Fig. S2). Given the potential link between mitochondrial biology and muscle protein metabolism and growing interest in the potential anabolic effects of dietary n-3 PUFAs, we also measured mixed muscle protein synthesis, reflecting the average synthesis rate of all muscle proteins over a 6-week labeling period with deuterium oxide. We found no differences in mixed muscle protein synthesis between young and old control mice, and there were no effects of EPA or DHA in old mice (Fig. 4B). Interestingly, young mice treated with DHA exhibited significantly higher mixed muscle FSR compared with young control mice (Fig. S2). These data show that although there appear to be some anabolic effects of DHA in young mice, neither EPA nor DHA stimulate mitochondrial or mixed muscle protein synthesis in old mice. Therefore, the RNA sequencing and stable isotope experiments demonstrate that EPA and DHA do not stimulate mitochondrial biogenesis in skeletal muscle.

### Eicosapentaenoic acid improves mitochondrial protein quality

In the absence of any apparent effects of EPA or DHA on muscle mitochondrial biogenesis or abundance, we determined whether the partial restoration of mitochondrial function by EPA in old mice could be explained by improved quality of the mitochondrial proteome. The rationale for this hypothesis was based on evidence that n-3 PUFAs may have antioxidant properties (Richard *et al*., 2008) or induce the expression of endogenous antioxidants (Lanza *et al*., 2013). Mitochondrial protein quality was evaluated using an unrestricted survey of post-translational modifications (PTMs) of the mitochondrial proteins that were detected by mass spectrometry. The most abundant PTMs detected were oxidation, deamidation, carbamylation, and acetylation (Table1). Of these modifications, carbamylation was significantly decreased with EPA treatment in old mice. Other PTMs such as oxidation and acetylation also exhibited modest, but nonsignificant trends for lower abundance in EPA-treated old mice (Table1). The lower abundance of protein PTMs points to improved quality of mitochondrial proteins as a potential mechanism underlying the restoration of mitochondrial function in old mice fed EPA. Because protein quality was improved with EPA treatment, we evaluated protein degradation by measuring the abundance of semitryptic peptides, a qualitative measure of *in vivo* proteolytic activity. There were no differences in semitryptic peptides between groups (Table1), indicating that the improvements in protein quality with EPA treatment are unlikely related to enhanced degradation and clearance of damaged proteins. There were no effects of EPA or DHA on PTMs or semitryptic peptides in young mice (Table S3).

**Table 1:** Post-translational modifications of mitochondrial proteins are less abundant with eicosapentaenoic acid

PTM	Young CON	Old CON	Old EPA	Old DHA
Oxidation	1310.5 ± 21.6	1329.2 ± 13.4	1308.5 ± 6.5	1315.8 ± 13.4
Deamidation	87.3 ± 2.8	90.8 ± 2.7	91.2 ± 2.4	85.0 ± 2.2
Carbamylation	25.3 ± 1.7	29.3 ± 1.2	20.2 ± 2.4*	24.7 ± 1.6
Acetylation	70.5 ± 4.6	82.5 ± 2.9	75.5 ± 3.9	78.8 ± 3.2
Semitryptic Peptides	355.3 ± 17.9	365.8 ± 4.4	345.3 ± 11.1	351.7 ± 5.1

Blind detection of post-translational modifications (PTMs) of mitochondrial proteins including semitryptic peptides was performed using mass spectrometry in muscle tissues (*n* = 6 per group). To compare PTM abundance across groups, spectral counts were normalized to total mitochondrial proteins and compared using a one-way ANOVA with a Tukey post hoc test. *P* < 0.05

* significant difference from old control.

## Discussion

This study shows that the age-related impairments in skeletal muscle mitochondrial oxidative capacity and efficiency are attenuated by supplementation with EPA but not DHA. Furthermore, this attenuation occurred without stimulating mitochondrial biogenesis, evidenced by the absence of any effect of EPA or DHA on mtDNA abundance, mitochondrial protein expression, or the fractional synthesis rates of mitochondrial proteins. Rather, this study provides evidence that EPA improved mitochondrial protein quality, which is likely to be an important factor in maintaining the function of the organelle in old mice.

There is growing interest in the influence of n-3 PUFAs on mitochondrial biology following early reports that n-3 PUFAs may stimulate mitochondrial biogenesis. For example, EPA supplementation activated PGC-1α and the downstream transcription factor TFAM in glioma cells (Jeng *et al*., 2009). Similarly, EPA and DHA increased PGC-1α and nuclear respiratory factor 1 (NRF1) in epididymal fat (Flachs *et al*., 2005) and skeletal muscle (Lanza *et al*., 2013) of mice. In spite of this promising evidence from gene expression measurements, much less is known about the effects of n-3 PUFAs on mitochondrial function. A recent report showed that EPA and DHA supplementation (3 g/day) for 12 weeks in young, healthy humans did not improve skeletal muscle mitochondrial capacity, but did increase mitochondrial sensitivity to ADP (Herbst *et al*., 2014). Several other reports (Yamaoka *et al*., 1988; Khairallah *et al*., 2012), including our own previous work (Lanza *et al*., 2013), failed to demonstrate any enhancement of mitochondrial content or function in response to n-3 PUFA supplementation. The current study shows a partial reversal of an age-associated decline of mitochondrial function by EPA in the quadriceps muscle, a mixed fiber type, of old mice (Fig.1A–D). The apparent discrepancy between the current finding and precedent literature may be explained by the possibility that EPA may enhance mitochondrial function only under conditions where dysfunction is evident. Indeed, in the current study, we found no improvements in mitochondrial function when EPA was given to young mice, but EPA improved mitochondrial function in older mice who exhibited ∼50% reduction in mitochondrial capacity compared with young. Consistent with the previous evidence that n-3 PUFAs decrease the apparent *k*_m_ of ADP in mitochondria (Herbst *et al*., 2014), we also find that EPA improved bioenergetic efficiency evidenced by increased respiratory control ratios (i.e., decreased proton leak). Improved bioenergetic efficiency and increased oxidative capacity without increases in mitochondrial content together indicate that EPA enhances the intrinsic function of mitochondria.

We provide new mechanistic insight into the biological effects of EPA and DHA through large-scale gene transcript profiling by RNA sequencing, untargeted quantitative proteomics, and direct measurements of *in vivo* muscle protein synthesis rates. Based on earlier observations that n-3 PUFAs activate a transcriptional pattern consistent with mitochondrial biogenesis (Jeng *et al*., 2009), we hypothesized that EPA and DHA would increase the expression of mitochondrial genes and proteins and increase the fractional synthesis rates of mitochondrial proteins in skeletal muscle of old mice. Although we confirmed that EPA increased *ppargc1a* mRNA levels, the genes and proteins involved in mitochondrial bioenergetics remained largely unchanged or were further downregulated, a finding inconsistent with the notion that EPA and DHA activate mitochondrial biogenesis. Further confirmation was achieved using *in vivo* labeling of muscle proteins to determine whether EPA or DHA increased the translational rate of mitochondrial proteins. The 25% reduction in mitochondrial protein synthesis in muscle of old control mice is similar to our previous report (Lanza *et al*., 2012); however, neither EPA nor DHA increased mitochondrial protein synthesis in old mice. These findings, combined with gene expression and protein expression measurements, demonstrate that EPA and DHA do not stimulate mitochondrial biogenesis in spite of effects of EPA on mitochondrial oxidative capacity and efficiency in old mice.

Having ruled out mitochondrial biogenesis as a potential mechanism underlying the effects of EPA on mitochondrial function, we discovered that EPA reduced deleterious post-translational modifications, most notably carbamylation of mitochondrial proteins. Carbamylation is a protein modification that is well characterized as a dominant mechanism within human atherosclerotic lesions, affecting the function of LDL receptor proteins (Wang *et al*., 2007). The modification is catalyzed by myeloperoxidase (MPO), which is most abundant in neutrophils and has been found within skeletal muscle under conditions of neutrophil infiltration in response to inflammation (Belcastro *et al*., 1996; Tiidus & Bombardier, 1999). As systemic low-grade inflammation is a hallmark of aging (Franceschi & Campisi, 2014) and n-3 PUFAs are known to have potent anti-inflammatory properties, it stands to reason that the decreased carbamylation of mitochondrial proteins in old EPA-treated mice may be consequent to reduced inflammation. Indeed, *acute phase response signaling* was the top downregulated canonical pathway in EPA-treated old mice compared with old control mice when we performed an unrestricted pathway analysis combining RNA and protein expression together (Table2). The possibility that anti-inflammatory effects of EPA may be driving improvements in skeletal muscle mitochondrial function is a possibility that requires further investigation.

**Table 2:**
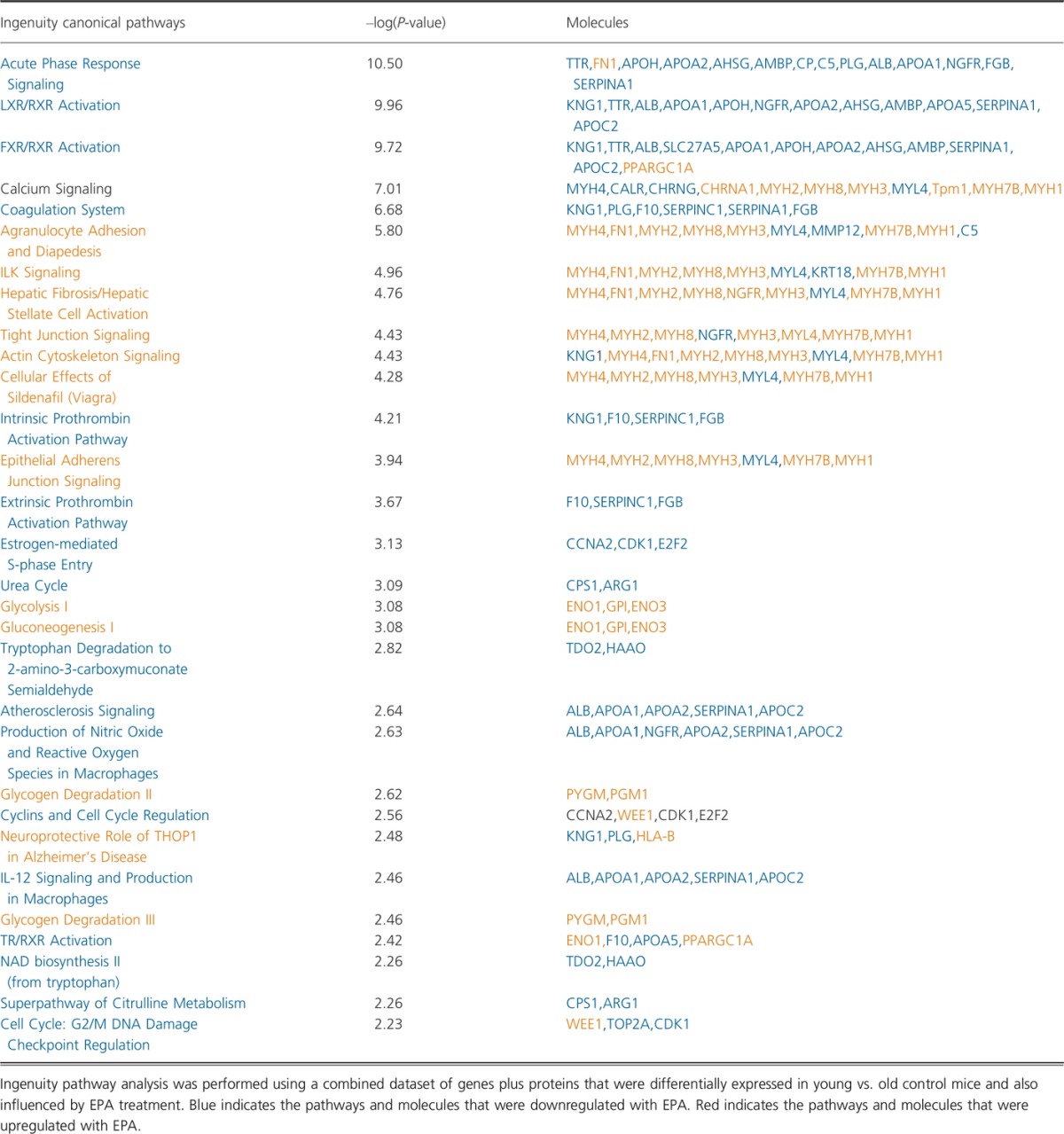
Canonical pathways affected by eicosapentaenoic acid (EPA) supplementation in old mice

In addition to our primary objective of evaluating the effects of EPA and DHA on mitochondrial biology in muscle of old mice, we also sought to elucidate potentially distinct biological effects of these two fatty acids on skeletal muscle. The motivation for this additional exploratory investigation stems from the abundance of literature where the effects of EPA and DHA are studied together in spite of evidence that they may be distinct in their actions (Rahman *et al*., 2008; Kamolrat & Gray, 2013; Wang *et al*., 2013). Consistent with this notion, we report that EPA, not DHA, partially restored mitochondrial oxidative capacity in skeletal muscle of old mice. As adequate mitochondrial ATP production is necessary to fuel the energetic demands of muscle protein synthesis, we determined whether the increases in mitochondrial oxidative capacity with EPA was accompanied by changes in mixed muscle protein synthesis, which have been previously reported to be decreased with age (Henderson *et al*., 2008). We found that neither EPA nor DHA increased mixed muscle protein synthesis in old mice; however, DHA significantly increased FSR in young mice. The possibility that n-3 PUFAs may attenuate sarcopenia by providing an additional anabolic stimulus to aging skeletal muscle is of high potential impact given that older adults exhibit blunted hypertrophic responses to resistance exercise training (Kosek *et al*., 2006) and attenuated increases in muscle protein synthesis in response to acute bouts of resistance exercise (Kumar *et al*., 2009; Fry *et al*., 2011). Several reports show that dietary n-3 PUFAs increase skeletal muscle protein synthesis in young and middle-aged adults (Smith *et al*., 2011b) as well as older adults (Smith *et al*., 2011a). Here, we sought to compare the effects of EPA and DHA, individually, on mixed muscle protein synthesis using deuterium oxide labeling *in vivo*. Although these findings appear to be in contrast with previous human intervention studies, Smith and colleagues found that n-3 PUFAs only increased FSR under postprandial conditions where insulin and amino acids were clamped at a high level for 3 h, but not in the postabsorptive state (Smith *et al*., 2011a,b). In the current study, we measured protein synthesis over a 6-week period that encompassed the entire continuum of fasting and fed states. It is therefore possible that the stimulatory effects of n-3 PUFAs exist in the postprandial state but are less evident in older mice when measured over a longer period of time that includes a range of fed and fasted states. Neither EPA nor DHA altered glucose tolerance or insulin sensitivity in young or old mice (Fig. S3).

Finally, EPA and DHA exhibited similar influence on genes and proteins related to the coagulation system, anti-inflammatory responses, and downregulation of the binding of retinoid acid receptor (RXR) to the bile acid and liver X receptors (FXR and LXR) (Tables2 and 3). Due to the small size of RXR pool, its dimerization with nuclear peroxisome proliferator-activated receptor (PPAR) is favored, leading to further activation of PPARGC1A. Additionally, DHA seems to affect a higher number of elements and pathways within muscle cells compared with EPA.

**Table 3:**
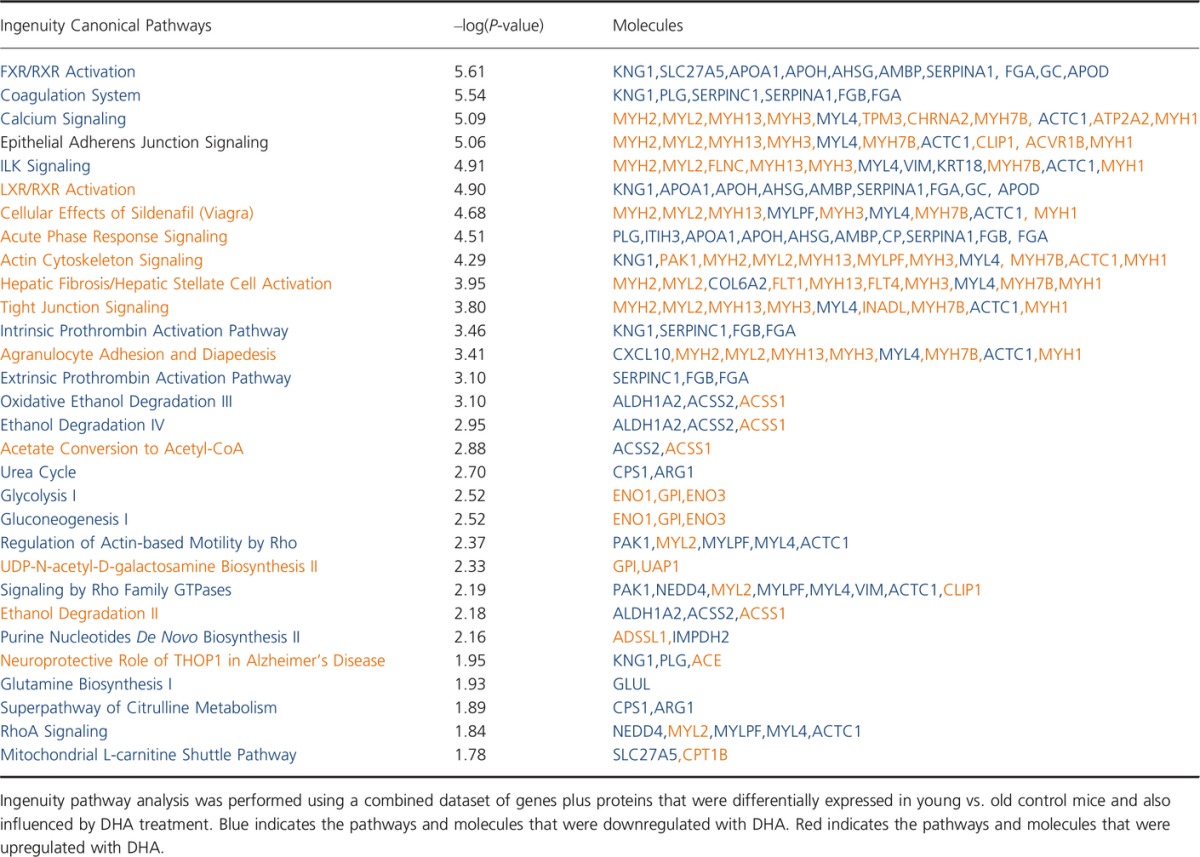
Canonical pathways affected by docosahexaenoic acid (DHA) supplementation in old mice

In conclusion, 10 weeks of supplementation with EPA partially restored skeletal muscle mitochondrial oxidative capacity by increasing the coupling efficiency of the mitochondrial electron transport chain and thereby improving the intrinsic function of mitochondria. Our results demonstrate that this partial restoration of oxidative capacity is not due to mitochondrial biogenesis, degradation, or changes in content. Instead, we propose a reduction in post-translational modifications, specifically carbamylation, to mitochondrial proteins is one mechanism that contributed to improved oxidative capacity after supplementation with EPA. An important next step will be to determine whether these observations in a rodent model of aging translate to therapeutic benefit in aging humans. Although humans rarely engage in activities that require the full energy-producing capacity of their mitochondria, the capacity of the organelle defines the functional reserve beyond the energetic needs of activities of daily living. It is conceivable that increasing the capacity and efficiency of mitochondria in the muscles of older adults could improve their ability to maintain a given level of muscle activity for extended periods of time (i.e., reduced muscle fatigue during activities of daily living). This possibility will require careful translational studies in humans with a focus on functional outcomes.

## Experimental procedures

### Animals

The Mayo Clinic Institutional Animal Care and Use Committee approved the protocol. We studied 36 adult (6 months) and 36 old (24 months) C57BL6 mice. Mice were randomly assigned to receive control chow (CON; 20% kcal protein, 70% kcal carbohydrate, 10% kcal fat) from Research Diets Inc. (New Brunswick, NJ, USA) or chow enriched with EPA or DHA (3.4% kcals) for 10 weeks. Diets were matched for macronutrient distribution (Table S4). Mice were housed in a temperature-controlled environment with a 12-h light–dark cycle and free access to food and water. Body weight and composition was measured at baseline and at 10 weeks by Echo-MRI (Fig. S4; Echo Medical Systems, Houston, TX, USA). All measurements were performed in quadriceps muscles.

### Mitochondrial energetics

Respiration of isolated mitochondria with glutamate + malate substrates and palmitoyl-L-carnitine substrates was performed as previously described (Lanza & Nair, 2009; Lanza *et al*., 2013). Briefly, mitochondria were isolated from fresh tissue by differential centrifugation. Respiration of isolated mitochondria was measured by high-resolution respirometry (Oxygraph, Oroboros Instruments, Innsbruck, Austria) using a stepwise protocol to evaluate various components of the electron transport system. Protein content of the mitochondrial suspension was measured using a colorimetric assay (Pierce 660-nm Protein Assay). Oxygen flux rates were expressed per tissue wet weight and normalized per milligram of mitochondrial protein. To evaluate the functional integrity of the isolated organelles, each sample run included a step where exogenous cytochrome c was added to the respiration chamber. In all experiments, the addition of cytochrome c did not augment respiration rates, indicating that the integrity of the mitochondrial outer membrane remained intact in these preparations.

### Protein fractional synthesis rates

Fractional synthesis rates was measured by two independent methods, first, by bolus injection and by long-term labeling with heavy water (^2^H_2_O). Detailed methods are outlined in Appendix S1 (Supporting information)**.**

### RNA sequencing

Total RNA was isolated using the TrueSeq method, and RNA libraries were prepared according to the manufacturer’s instructions for the TruSeq RNA Sample Prep Kit v2 (Illumina, Hayward, CA). Libraries were loaded onto paired end flow cells following the standard protocol for the Illumina cBot and cBot Paired end cluster kit version 3. Flow cells were sequenced as 51 × 2 paired end reads on an Illumina HiSeq 2000 using TruSeq SBS sequencing kit version 3 and hcs v2.0.12 data collection software. Base calling was performed using Illumina’s RTA version 1.17.21.3. The RNA-Seq data were analyzed using map-rseq v.1.2.1 (Kalari *et al*., 2014), the Mayo Bioinformatics Core pipeline. MAP-RSeq consists of alignment with tophat 2.0.6 (Kim *et al*., 2013, p.2) against the hg19 genome build and gene counts with the htseq software 0.5.3p9 (http://www.huber.embl.de/users/anders/HTSeq/doc/overview.html) using gene annotation files obtained from Illumina (http://cufflinks.cbcb.umd.edu/igenomes.html). Normalization and differential expression analysis were performed using edgeR 2.6.2 (Robinson *et al*., 2010).

### Mass spectrometry-based proteomics

We used quadriceps muscle tissue to compare the relative expression of proteins in each group of mice (*n* = 6 per group). Tissues were pulverized and sonicated in RIPA buffer and subjected to in-gel trypsin digestion. Peptides were identified using nano-LC-ESA-MS/MS and a two-step workflow to detect and quantify proteins, post-translational modifications and semitryptic peptides was followed as previously published (Lanza *et al*., 2012) and provided in detail in Appendix S1.

### Statistical analyses

Statistical analysis was performed using prism v6.0e (GraphPad Software Inc, La Jolla, CA, USA). Differences between age (Young vs. Old) and treatment (CON, EPA, DHA) were compared using a two-way analysis of variance (ANOVA). When a significant interaction was detected, post hoc analysis was performed with the Tukey’s procedure. Significance was set at *P* ≤ 0.05. Data are presented as means ± SEM.

## Funding

Funding for this work was provided by a grant from the American Federation for Aging Research (I.R.L.), the Mayo Clinic Center for Cell Signaling in Gastroenterology NIDDK P30DK084567, T32 DK007198 (M.L.J), KL2 TR000136-07 (M.L.J.), and UL1 TR000135.

## Conflict of interest

The authors have declared that no conflict of interest exists.

## Author contributions

MLJ, AZL, SD, and IRL performed data analysis and wrote the manuscript. MF and MKH performed alanine enrichment measurements for muscle protein synthesis. IRL conceived the experiments and performed data collection.

## Supporting Information

**Fig. S1** EPA and DHA do not affect muscle mitochondrial respiratory function in young mice. **Fig. S2** EPA and DHA do not affect mitochondrial protein synthesis in muscle from young mice. **Fig. S3** Glucose tolerance was measured from intraperitoneal glucose tests (A,B). **Fig. S4** At baseline (0 wk) mice were randomly assigned to either control, EPA or DHA treatment. **Table S1** Individual proteins from each of the canonical pathways included in Figure 2. **Table S2** Individual genes from each of the canonical pathways included in Figure 3. **Table S3** Posttranslational modifications in young mice. **Table S4** Composition of purified diets **Appendix S1** Experimental procedures.

** **

** **
