# Supplementary material for: Eicosapentaenoic acid but not docosahexaenoic acid restores skeletal muscle mitochondrial oxidative capacity in old mice
Source: Aging Cell. 2015 May 25;14(5):734–43. doi: 10.1111/acel.12352 (PMC4568961; doi:10.1111/acel.12352)
Supplement: Supplementary file 1 [file acel0014-0734-sd1.docx]

**Supplemental Information**

**EPA but not DHA restores skeletal muscle mitochondrial oxidative capacity in old mice**

Matthew L. Johnson, Antigoni Z. Lalia, Surendra Dasari, Maximilian Pallauf, Mark Fitch, Marc K. Hellerstein, Ian R. Lanza

**Inventory of Supplemental Information**

**Figure S1,** related to Figure 1: This figure shows skeletal muscle mitochondrial function measured in young mice (n=8 per group) with control, EPA, or DHA supplementation. These data provide support that neither EPA nor DHA impact mitochondrial function in young mice.

**Figure S2,** related to Figure 4: This figure shows mitochondrial and whole muscle fractional synthesis rates in young mice (n=8 per group) with control, EPA, or DHA supplementation. These data provide support that neither EPA nor DHA stimulate skeletal muscle mitochondrial protein synthesis, but do support an effect of DHA on whole muscle synthesis rates.

**Figure S3:** This figure shows glucose tolerance and insulin sensitivity measured in young and old mice fed control chow, EPA or DHA at baseline and 10 weeks.

**Figure S4:** This figure shows body weight and body composition for young and old mice at baseline and after 10 weeks of intervention.

**Table S1, related to Figure 2:** This table shows the individual proteins from each canonical pathway included in Figure 2 provided by Ingenuity Pathway Analysis.

**Table S2, related to Figure 3:** This table shows the individual proteins from each canonical pathway included in Figure 3 provided by Ingenuity Pathway

**Table S3, related to Table 1:** This table shows the posttranslational modifications in young mice after 10 weeks of either control, EPA, or DHA supplementation.

**Table S4:** This table shows the dietary composition of the control, EPA, and DHA diets used during the study.

**Figure S1. EPA and DHA Do Not Affect Muscle Mitochondrial Respiratory Function In Young Mice**

Respiration rates were with glutamate + malate substrates were measured in isolated mitochondria (A & B) with substrates targeting complex I (CI), complex I+II (CI+II), and complex II (CII). Nonmitochondrial oxygen consumption was measured in the presence of antimycin A (AA). Respiration rates were also measured using palymitoyl-carnatine substrates (C & D). Respiration rates were expressed per tissue wet (A & C) and mitochondrial protein content (B & D). Respiratory control ratio (RCR, state 3/state 4) and phosphorylation efficiency (ADP:O) were measured in isolated mitochondria (E & F). Bars represent means ± SEM. n = 8 per group.

**Figure S2.** EPA and DHA do not affect mitochondrial protein synthesis in muscle from young mice (A) , but DHA increases mixed muscle protein synthesis in young mice (B). Fractional synthesis rates of mitochondrial proteins was measured *in vivo* from the rate of incorporation of ^13^C_6_ phenylalanine and mass spectrometry (A). The overall mixed muscle protein synthesis rate was measured by long-term labeling with deuterium oxide (B). Bars represent means ± SEM. *, significant statistical differences from young control (p < 0.05, Tukey’s HSD). n = 8 per group.

**Figure S3.** Glucose tolerance was measured from intraperitoneal glucose tests (A,B). The area above baseline (AAB) for glucose tolerance tests was similar across groups at baseline (0 weeks, C), and did not differ significantly after 10 weeks of feeding (D,E). Insulin sensitivity was measured by intraperitoneal insulin tolerance tests (F,G). The area above the curve below baseline (AACBB) was similar in all groups at baseline (H). After 10 weeks of feeding, AACBB increased in all groups except for EPA-treated old mice (I,J). Data are means ± SEM. * denotes significantly (p<0.05) different from young control.

**Figure S4.** At baseline (0 wk) mice were randomly assigned to either control, EPA or DHA treatment. Body mass (A), lean mass (B), fat mass (C) and percent body fat (D) were measured at baseline and after 10 weeks. Bars represent means ± SEM. Groups not connected by the same letters are significantly different (p < 0.05, Tukey’s HSD). Post hoc comparisons were not made when the overall group effect was not significant. n = 8 per group.

**Supplemental Table 1: Individual proteins from each of the canonical pathways included in Figure 2.**

**Supplemental Table 2: Individual genes from each of the canonical pathways included in Figure 3.**

**Supplemental Table 3: Posttranslational Modifications In Young Mice**

| PTM | Young CON | Young EPA | Young DHA |
| --- | --- | --- | --- |
| Oxidation | 1310.5 ± 21.6 | 1331.8 ± 13.5 | 1322.5 ± 14.2 |
| Deamidation | 87.3 ± 2.8 | 91.3 ± 3.1 | 86.2 ± 2.4 |
| Carbamylation | 25.3 ± 1.7 | 27.3 ± 2.9 | 25.3 ± 1.7 |
| Acetylation | 70.5 ± 4.6 | 80.0 ± 4.2 | 80.0 ± 1.7 |
| Semitryptic  Peptides | 355.3 ± 17.9 | 370.7 ± 7.0 | 368.2 ± 9.5 |

Blind detection of posttranslational modifications (PTMs) of mitochondrial proteins including semitryptic peptides was performed using mass spectrometry in muscle tissues (n = 6 per group). To compare PTM abundance across groups, spectral counts were normalized to total mitochondrial proteins, and compared using a one-way ANOVA.

**Supplemental Table 4:** composition of purified diets

**SUPPLEMENTAL EXPERIMENTAL PROCEDURES**

*Protein fractional synthesis rates (FSR).* FSR was measured by two independent methods. First, by bolus injection as previously reported (You et al. 2009). Briefly, on the morning of the FSR measurements food was removed 3 hours before intraperitoneal injection of [*ring*-^13^C_6_] phenylalanine (Cambridge Isotope Laboratories, Cambridge MA, 99% molar percent excess, 0.015 mg/g bodyweight). 20 minutes after injection mice were sacrificed and individual skeletal muscle tissues were snap frozen in liquid nitrogen after blotting for blood and removing visible fat and connective tissue. All samples were stored at -80C. Total mixed muscle, mitochondrial, and tissue-free fluid fractions were isolated from the same quadriceps muscle. Protein samples were hydrolyzed for 24 hours in 0.05 mol/L HCl at 110 C in the presence of cation-exchange resin (AG 50W-X8, BioRad Laboratories, Hercules, CA) and resultant amino acids were purified over a column of the same resin. Molar percent excess (MPE) of mixed muscle, mitochondrial, and tissue-free-fluid fractions were then measured by liquid chromatography tandem mass spectrometry (LC/MS/MS) as previously described (Jaleel et al. 2008).

Second, mixed muscle FSR was measured by long-term labeling with heavy water (^2^H_2_O) as previously reported (Miller et al. 2012). Briefly, after intraperitoneal injection of 99% MPE ^2^H_2_O calculated to enrich the body water pool to 5% animals were then allowed to drink *ad lib* 4% enriched water for 6 weeks. Skeletal muscle tissue was harvested as described above. After homogenization tissue was hydrolyzed by incubation in 6 N HCl at 120 C for 24 hours and subsequent amino acids passed through a cation ion-exchange column. Vacuum dried samples were then suspended in 50% acetonitrile, 50mM K_2_HPO_4_, pH 11 for derivitization by pentafluorobenzyl bromide (Pierce Scientific, Rockford, IL). Mass-to-charge ratios of 448, 449, and 450 were monitored for the pentafluorobenzyl-*N,N*-di(pentaflurobenzyl)alaninate derivative, which contain the primary daughter ions of the original hydrocarbon bonds of alanine. The newly synthesized fraction (*f*) of mixed muscle was calculated from the precursor enrichment (*p*) using mass isotopomer distribution analysis (MIDA) and expressed as the percent of proteins newly synthesized over the entire labeling period (%FSR) (Busch et al. 2006).

*Mass Spectrometry-based Proteomics.*

We used quadriceps muscle tissue to compare the relative expression of proteins in each group of mice (n = 6 per group). Tissues were pulverized and sonicated in RIPA buffer. Samples were prepared for SDS-PAGE at a final protein concentration of 1 mg/ml. Samples (15 mg/well) were resolved on 4%–12% NuPAGE Novex Bis-Tris Midi gels after which each lane was cut into 8 sections to reduce sample complexity during mass spectrometry analysis. Following in-gel trypsin digestion, peptides were identified and quantified using nano-flow liquid chromatography electrospray tandem mass spectrometry (nanoLC-ESI-MS/MS) using a QExactive Mass Spectrometer (Thermo Fisher Scientific, Waltham, MA) coupled to an Eksigent nanoLC-2D with an AS1 autosampler (Eksigent, Dublin, CA).

A portion of each peptide mixture was loaded via an autosampler onto a 0.25 uL Optipak trap (New Objective, Woburn, MA) custom packed with 5 um Magic C8 stationary phase (Michrom Bioresources, Auburn, CA). After loading and washing, the pre-column trap was placed in-line with a 34 cm by 0.075 mm spray tip packed in-house with 3 um Magic C18 stationary phase (Michrom Bioresources, Auburn, CA). Peptides were separated and eluted over a 60-minute gradient of 2 to 40% mobile phase B where mobile phase A was water/acetonitrile/formic acid (98/2/0.1 by volume) and mobile phase B was acetonitrile/isopropanol/water/formic acid (80/10/10/0.1 by volume). QExactive survey scans were acquired at 70,000 resolving power using a target ion population of 1e6 charges followed by top 12 data-dependant MS/MS scans at 17,500 resolving power with a target ion population of 5e5 charges, isolation window of 2 Da, and normalized collision energy at 26. Precursor masses selected for tandem MS experiments were placed on an exclusion list for 45 seconds. A total of approximately 12.9 million MS/MS were collected from all LC-MS/MS analyses. Binary spectral data present in the raw files were converted to mzML formats using the msConvert tool of the ProteoWizard library (Kessner et al. 2008).

*Bioinformatics Methods*

Protein Identification. The tandem MS/MS spectra present in all the data files were matched against a composite protein sequence database using MyriMatch (version 2.1.120) search engine. This database contained protein sequences from the RefSeq database (release 53) sub-selected for human species and also common contaminants (wool, cotton, etc.). Human mitochondrial protein sequences were extracted from SwissProt database (version 2013_05) and added to the database. Reversed sequence entries were appended to the database for estimating peptide identification false discovery rates (FDRs). MyriMatch was configured to derive semitryptic peptides from the sequence database while searching for carbamidomethylation of cysteine (+57.0125 Da) as static modification. The software also considered the oxidation of methionine, tryptophan and tyrosine residues (+15.996 Da) and formation of N-terminal pyroglutamine (-17.0265 Da) as variable modifications. The software allowed up to 3 missed cleavage sites while deriving the peptides and assumed 10ppm m/z error on both precursor and fragments while performing the peptide-spectrum matching. IDPicker software (version 3.0.515) filtered the peptide identifications at 2% FDR. The software automatically combined the MVH, mzFidelity, and XCorr scores for the q-value filtering. Filtered peptide identifications were assembled into protein identifications following parsimony rules. Protein identifications with at least two distinct peptide identifications were considered for further analysis.

Label-free Protein Differential Expression. The number of MS/MS spectra matching to a protein was considered as a semi-quantitative measure of its abundance (Li et al. 2010; Liu et al. 2004). We utilized this spectral counting-based method for detecting differentially expressed proteins between sample groups. For this, IDPicker software combined the protein and peptide identifications from all of the samples. Proteins with at least 10 MS/MS matches were considered for the analysis, which resulted in an overall protein FDR of 2%. Resulting protein identifications and corresponding spectral counts were grouped by the sample and its group (combination of EPA and DHA with each of the control, young and old cohorts). Spectral abundance of each protein was compared across appropriate groups using QuasiTel software (Li et al. 2010). QuasiTel software was configured to utilize the biological replicates when comparing samples of two groups. For each paired group comparison, proteins that had a Quasi P-value of ≤ 0.05 and log2 fold change of at least 0.5 were considered for pathway analysis.

Label-free Global Quantification of Semitryptic Peptides: This method utilized the same protein and spectral matches that were utilized in the above analysis. The differential abundance of mitochondrial semitryptic peptides between sample groups was considered as a semi-quantitative measure for in vivo degradation of mitochondrial proteins. To measure this, we summed the total number of spectra matching to mitochondrial semitryptic peptides detected in all samples in each study group and normalized it to the total number of spectra matching to all mitochondrial peptides in that same group. The normalized spectral counts of mitochondrial semitryptic peptides between the two study groups were compared in a pairwise fashion using chi-square test p value cut-off of P<0.05 and fold-change equal to or greater than 0.20. The fold change of the mitochondrial semitryptic peptides between the two study groups was calculated using the RSC formula in reference (Old et al. 2005).

Global Identification and Quantification of Posttranslational Modifications (PTMs). We utilized a three-step approach for detecting and quantifying the PTMs present in the samples. In the first step, we searched the MS/MS data for an unrestrictive set of modifications (Dasari et al. 2011). For this, a subset protein sequence database was created using the confident proteins identified in the semitryptic peptide quantification step. DirecTag software (Tabb et al. 2008) derived short sequence tags of three amino acids in length from the MS/MS. TagRecon (Dasari et al. 2011) software matched the inferred tags to the subset database. The software was configured to derive semitryptic peptides from the database and look for any unexpected mass shifts in the peptides. TagRecon also looked for the above described static and variable modifications as well. IDPicker software filtered the resulting peptide identifications as described above. PTMDigger software (Dasari et al. 2011) detected the most abundant modifications detected in the samples following the “blind PTM” search result attestation procedure described elsewhere (Dasari et al. 2011). The following were the list of confident PTMs detected in all samples groups: oxidation (on methionine, phenylalanine and tryptophan), deamidation (on asparagine and glutamine) and carbamylation (on methionine), acetylation (on lysine) modifications. In the second step, these high-confident modifications were fed into a “targeted PTM” search (Dasari et al. 2011). For this, TagRecon configuration was changed to re-analyze the MS/MS spectra using the preferred PTMs instead of an unrestrictive PTM set. IDPicker filtered the peptide identifications and assembled them into protein identifications following the above described protocol. Proteins with at least two unique peptide identifications and 10 MS/MS matches over all samples were considered for PTM quantification analysis in the third step. Candidate PTM’s abundance in each sample was independently estimated by using the total number of spectra matching to the mitochondrial peptides containing that modification. Each PTM’s abundances in all samples of a study group were summed and normalized to the total number of spectra matching to all mitochondrial proteins present in that study group. A PTM’s abundance between two study groups was compared using a fisher’s exact test. The fold change of a PTM’s abundance between two groups was computed using the RSC formula in the reference (Old et al. 2005).

*Glucose and insulin tolerance tests*

Mice were fasted for 6 hours with free access to water before the tests. For glucose tolerance tests, glucose was injected intraperitoneally (2mg/kg body weight) and blood glucose was measured from the tail vein at 5, 10, 15, 30, 60, and 120 minutes following glucose injection. For insulin tolerance tests, insulin was injected intraperitoneally and blood glucose was measured at 15, 30, 60, and 120 minutes following injection. Glucose tolerance tests were quantified from the integrated area above baseline of glucose. Insulin tolerance tests were quantified as the integrated area above the curve below baseline.

**SUPPLEMENTAL REFERENCES:**

Busch R, Kim Y-K, Neese RA, Schade-Serin V, Collins M, Awada M, Gardner JL, Beysen C, Marino ME, Misell LM & Hellerstein MK (2006) Measurement of protein turnover rates by heavy water labeling of nonessential amino acids. *Biochim. Biophys. Acta* 1760, 730–744.

Dasari S, Chambers MC, Codreanu SG, Liebler DC, Collins BC, Pennington SR, Gallagher WM & Tabb DL (2011) Sequence tagging reveals unexpected modifications in toxicoproteomics. *Chem. Res. Toxicol.* 24, 204–216.

Jaleel A, Short KR, Asmann YW, Klaus KA, Morse DM, Ford GC & Nair KS (2008) In vivo measurement of synthesis rate of individual skeletal muscle mitochondrial proteins. *AJP Endocrinol. Metab.* 295, E1255–E1268.

Kessner D, Chambers M, Burke R, Agus D & Mallick P (2008) ProteoWizard: open source software for rapid proteomics tools development. *Bioinformatics* 24, 2534–2536.

Li M, Gray W, Zhang H, Chung CH, Billheimer D, Yarbrough WG, Liebler DC, Shyr Y & Slebos RJC (2010) Comparative shotgun proteomics using spectral count data and quasi-likelihood modeling. *J. Proteome Res.* 9, 4295–4305.

Liu H, Sadygov RG & Yates JR (2004) A model for random sampling and estimation of relative protein abundance in shotgun proteomics. *Anal. Chem.* 76, 4193–4201.

Miller BF, Robinson MM, Bruss MD, Hellerstein M & Hamilton KL (2012) A comprehensive assessment of mitochondrial protein synthesis and cellular proliferation with age and caloric restriction. *Aging Cell* 11, 150–161.

Old WM, Meyer-Arendt K, Aveline-Wolf L, Pierce KG, Mendoza A, Sevinsky JR, Resing KA & Ahn NG (2005) Comparison of label-free methods for quantifying human proteins by shotgun proteomics. *Mol. Cell. Proteomics MCP* 4, 1487–1502.

Tabb DL, Ma Z-Q, Martin DB, Ham A-JL & Chambers MC (2008) DirecTag: accurate sequence tags from peptide MS/MS through statistical scoring. *J. Proteome Res.* 7, 3838–3846.

You YN, Short KR, Jourdan M, Klaus KA, Walrand S & Nair KS (2009) The effect of high glucocorticoid administration and food restriction on rodent skeletal muscle mitochondrial function and protein metabolism. *PloS One* 4, e5283.
