# Supplementary figures and images for: Eicosapentaenoic acid but not docosahexaenoic acid restores skeletal muscle mitochondrial oxidative capacity in old mice
Source: Aging Cell. 2015 May 25;14(5):734–43. doi: 10.1111/acel.12352 (PMC4568961; doi:10.1111/acel.12352)

0 weeks

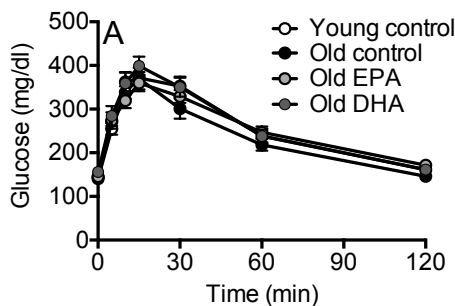

10 weeks

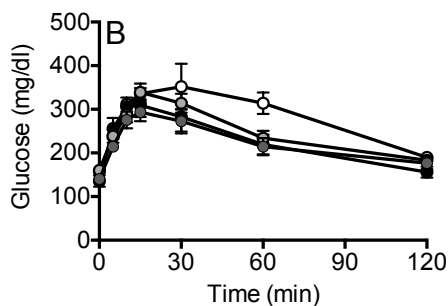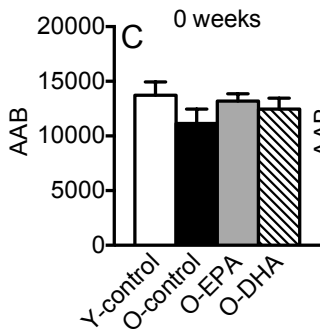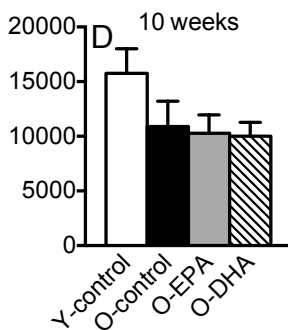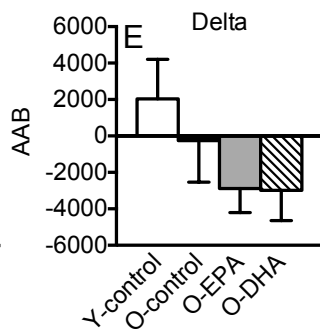

0 weeks

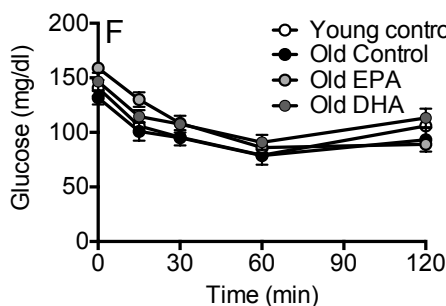

10 weeks

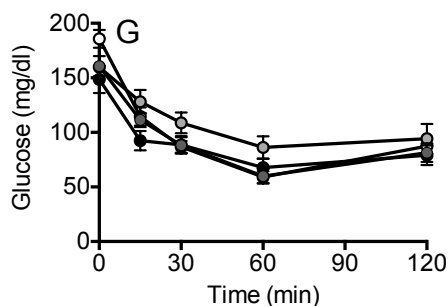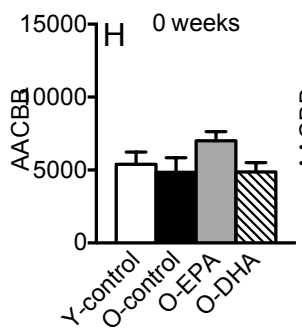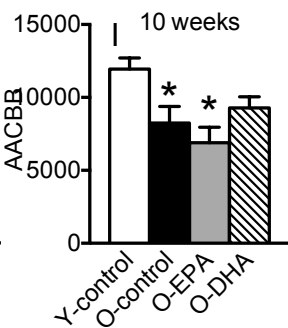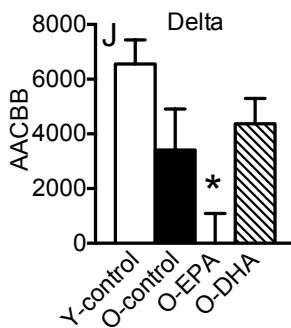

Supplement: Supplementary file 2 [file acel0014-0734-sd2.pdf]

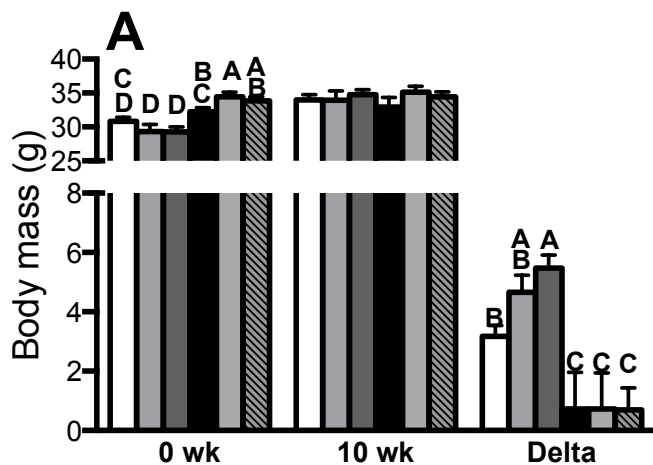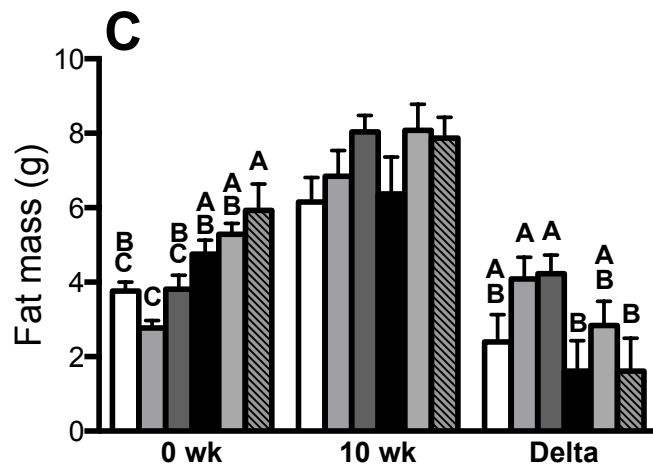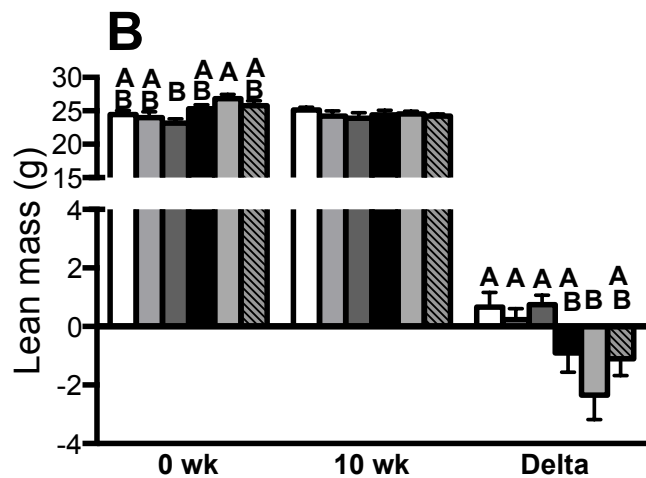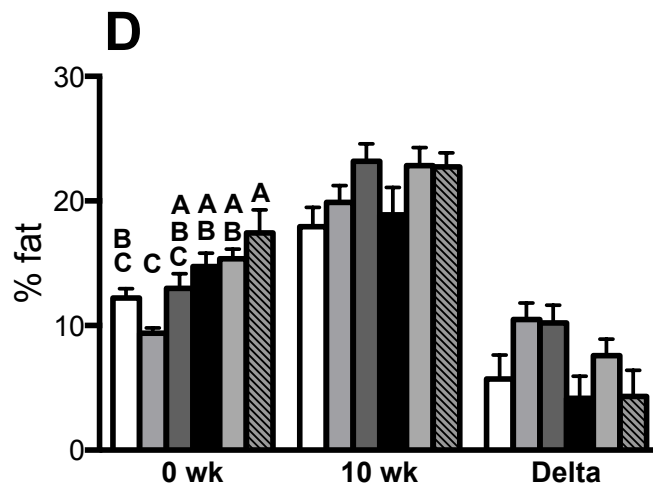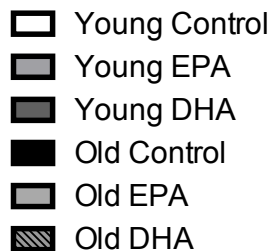

Supplement: Supplementary file 3 [file acel0014-0734-sd3.pdf]
